# Extracellular vesicles derived from antigen-presenting cells pulsed with foot and mouth virus vaccine-antigens act as carriers of viral proteins and stimulate B cell response

**DOI:** 10.3389/fimmu.2024.1440667

**Published:** 2024-08-08

**Authors:** Florencia Menay, Federico Cocozza, Maria J. Gravisaco, Analia Elisei, Javier Ignacio Re, Alejandra Ferella, Claudia Waldner, Claudia Mongini

**Affiliations:** ^1^ Laboratorio de Microvesículas, Exosomas y miRNA, Instituto de Virología y Innovaciones Tecnológicas (IVIT)-Consejo Nacional de Investigaciones Científicas y Técnicas (CONICET)-Instituto Nacional de Tecnología Agropecuaria (INTA), Hurlingham, Buenos Aires, Argentina; ^2^ Centro de Investigación en Ciencias Veterinarias y Agronómicas (CICVYA), Instituto Nacional de Tecnología Agropecuaria (INTA), Hurlingham, Buenos Aires, Argentina; ^3^ Institut National de la Santé et de la Recherche Médicale (INSERM-U932), Institut Curie, París, France; ^4^ Departamento de Patología, Servicio Nacional de Salud y Calidad Agroalimentaria (SENASA), Martinez, Buenos Aires, Argentina; ^5^ Laboratorio de Inmunología Celular y Molecular, Centro de Estudios Farmacologicos y Botanicos (CEFYBO) CONICET, Ciudad Autónoma de Buenos Aires, Argentina

**Keywords:** extracellular vesicles, foot and mouth disease virus, antiviral immune response, antigen presenting cells, B cell activation

## Abstract

Foot and mouth disease (FMD) is a highly contagious infection caused by FMD-virus (FMDV) that affects livestock worldwide with significant economic impact. The main strategy for the control is vaccination with FMDV chemically inactivated with binary ethylenimine (FMDVi). In FMDV infection and vaccination, B cell response plays a major role by providing neutralizing/protective antibodies in animal models and natural hosts. Extracellular vesicles (EVs) and small EVs (sEVs) such as exosomes are important in cellular communication. EVs secreted by antigen-presenting cells (APC) like dendritic cells (DCs) participate in the activation of B and T cells through the presentation of native antigen membrane-associated to B cells or by transferring MHC-peptide complexes to T cells and even complete antigens from DCs. In this study, we demonstrate for the first time that APC activated with the FMDVi O1 Campos vaccine-antigens secrete EVs expressing viral proteins/peptides that could stimulate FMDV-specific immune response. The secretion of EVs-FMDVi is a time-dependent process and can only be isolated within the first 24 h post-activation. These vesicles express classical EVs markers (CD9, CD81, and CD63), along with immunoregulatory molecules (MHC-II and CD86). With an average size of 155 nm, they belong to the category of EVs. Studies conducted *in vitro* have demonstrated that EVs-FMDVi express antigens that can stimulate a specific B cell response against FMDV, including both marginal zone B cells (MZB) and follicular B cells (FoB). These vesicles can also indirectly or directly affect T cells, indicating that they express both B and T epitopes. Additionally, lymphocyte expansion induced by EVs-FMDVi is greater in splenocytes that have previously encountered viral antigens *in vivo*. The present study sheds light on the role of EVs derived from APC in regulating the adaptive immunity against FMDV. This novel insight contributes to our current understanding of the immune mechanisms triggered by APC during the antiviral immune response. Furthermore, these findings may have practical implications for the development of new vaccine platforms, providing a rational basis for the design of more effective vaccines against FMDV and other viral diseases.

## 1 Introduction

The International Society for Extracellular Vesicles (ISEV) defines EVs as: “particles naturally released from the cell that are delimited by a lipid bilayer and cannot replicate, i.e. do not contain a functional nucleus” (1, 2). Although they were initially considered cellular waste bags, their role in multiple physiological and pathological processes is now undeniable. EVs participate in numerous biological processes, like antigen presentation and modulation of the immune response, intercellular communication, and transfer of molecules such as nucleic acids, proteins, and surface molecules. Specifically, EVs play a very important role in immune system communication being an alternative to the traditional routes of cell communication characterized by soluble factors or cell-cell contact (3, 4).

EVs secreted by both immune and non-immune cells perform important functions in immunity, by mediating immune stimulation or suppression and can lead to inflammatory, autoimmune, and infectious pathological diseases. A large part of the proteins expressed in EVs correspond with the proteins expressed in the cell of origin. In this way, changes that occur in the cell can be reflected in EVs. For example, when dendritic cells (DCs) mature and increase the expression of co-stimulatory molecules and MHC molecules, these changes also occur in EVs. Furthermore, EVs secreted by DCs differentiated from murine bone marrow (BMDC) and previously activated with LPS or IFN-γ have been shown to contain increased levels of MHC-II, CD86, and the adhesion molecule ICAM-I. Sometimes, the expression of molecules on EVs can explain their involvement in the regulation of the immune system (5–8).

Foot-and-mouth disease (FMD) is a highly contagious disease of worldwide livestock and has an important economic impact. The main strategy for the control is vaccination with foot-and-mouth disease-virus (FMDV) chemically inactivated with binary ethylenimide (FMDVi). Today, in Argentina, the FMDV National Eradication Plan implements the vaccine containing strains O1 Campos, A24 Cruzeiro, A Argentina 2001, and C3 Indaial, which are the ones circulating in Argentina (9). It has been demonstrated that FMDVi elicits an effective immune response and protects against infection without establishing sustained immunity over time and requires regular re-inoculations. In FMDV infection and vaccination, B cell response plays a major role by providing neutralizing/protective antibodies in both animal models and natural hosts. Our results demonstrated that APC internalize the vaccine antigens FMDVi and become activated. This activation is reflected as an increased expression of maturation markers such as MHC-II and CD86 molecules. As a result of this interaction, activated APC release sEVs expressing FMDVi proteins/peptides (sEVs- FMDVi), either MHC-associated or membrane-associated, due to antigen redirection to the sEVs within the DCs. Further, these EVs derived from APC previously pulsed with the antigens included in the commercial vaccine against FMDV modulate the antigen-specific immune response favoring an improved host antiviral immunity.

## 2 Materials and methods

### 2.1 Mice

Six-to-ten-week-old female immunocompetent BALB/c mice were purchased from the School of Veterinary Sciences, Universidad de Buenos Aires (Buenos Aires, Argentina). Animals were fed on Cargill pellets and water *ad libitum*. The entire work was performed using the murine model used for the study of the immune response against FMDV previously developed and detailed by other groups (10–13). All animal procedures were performed following the “Guide for the use and care of experimental animals” and approved by the Institutional Animal Care and Use Committee of Instituto Nacional de Tecnología Agropecuaria (INTA) (ethic code: 28/2016).

### 2.2 Inactivated virus and monovalent FMD vaccine

The O1 Campos strain was selected for this study because it conforms to the antigen banks that are used in case of FMDV outbreaks around the world (14) The O1 Campos FMDV was kindly provided by Biogénesis Bagó S.A. laboratory as a binary ethyleneimine inactivated virus suspension (FMDVi) (15). For *in vitro* assays, only sucrose gradient purified FMDVi was used (16). The monovalent vaccine for FMDV O1 Campos was also kindly provided by Biogénesis Bagó S.A laboratory and verified for sterility, safety, and purity according to SENASA Resolution (No. 609/2010).

### 2.3 Differentiation of APC

Bone marrow cells were isolated from female BALB/c mice between 6 and 10 weeks of age by flushing femurs with complete medium (RPMI 1640 supplemented with 10% FCS-EVs-depleted, 2mM glutamine, 25mM HEPES buffer, 100 IU/ml penicillin, 100 μg/ml streptomycin, 0.05 mM 2-mercaptoethanol and 10 U/ml Polymyxin B). Ten million bone marrow cells were cultured in Petri dishes in a complete medium with recombinant GM-CSF (20 ng/ml, Peprotech). On day 3, 10 ml of fresh culture medium with 40 ng/ml rGM-CSF was added to each Petri dish. On day 6, half of the medium was removed, and the cell suspension was centrifuged at 300 xg for 5 min, the pellet was resuspended in a new medium supplemented with rGM-CSF (40 ng/ml) and added to the culture (17). The morphology of the cells was monitored on a daily basis. At days 5–6, the cells exhibited the typical characteristics of immature dendritic cells, including the formation of cells clusters and dendrites, an irregular morphology, and a mixture of attached and suspension cells. On day 8, non-adherent cells and loosely adherent cells were harvested, pooled, and used for the experiment. The average yield of differentiation was 7.86 x 10^6^ ± 2.43 bone marrow dendritic cells (BMDC) from 10.00 x 10^6^ precursors. A population of cells with viability higher than 85% was obtained. Based on the presence of CD11b and F4/80 molecules and their morphological characteristics (data not shown), these APC could be included within the subpopulations of inflammatory or conventional DCs. However, taking into consideration the heterogeneity of the cells obtained, it was decided to classify them as APC in general. The population of cells obtained on day 8 of culture expressed MHC class II (>85%), F4/80, CD86, CD11b, and CD11c, but no Gr-1 (**Supplementary Figure S1**).

### 2.4 *In vitro* APC pulse load with BEI-FMDVi: determination of optimal virus concentration, APC pulsing time, and EVs collection time

10x10^6^ APC were incubated for 16 hours with 1, 5, and 10 μg of purified BEI-FMDVi. After this time, the cells were washed twice to remove antigen remnants and then cultured for 24 hours in complete RPMI medium. As a negative control, APC cultured in the presence of RPMI medium only was used. As a positive control of activation, APC incubated with 5 μg/ml of LPS or with POLI I:C 50 µg/ml were used (18). The pulsing time and EV collection time was determined using ten million APC incubated with 10 µg/ml of purified non-infectious BEI-FMDVi serotype O1 Campos for 16 or 4 h at 37°C in 6-well tissue-culture plates of complete medium. Non-stimulated APC were used as negative control (RPMI). APC with 5 µg/ml LPS was used as a positive control of maturation. For the isolation of EVs, APC were washed twice with complete medium and cultured in Petri dishes for 24 h or 36 h. The activation state of APC was evaluated by flow cytometry. The expression of classical activation markers such as MHC-II and CD86 was determined using specific monoclonal antibodies for each protein. For analysis, cells were gated by forward and side scatter as well as CD11c+ marker to eliminate the contribution of other populations such as granulocytes or macrophages that are not of interest.

### 2.5 FMDV internalization

The fluorescent labeling of the virus was accomplished using the procedures detailed by Harwood (19) with modifications. The label of virus was performed by mixing 1 mg of viral protein with 100 μg/ml FITC in 0.1 M bicarbonate buffer (pH 9.5) for 1 h at 37°C. Unconjugated dye was removed by dialyzed. APC were then incubated with 10 or 30 µg/ml FMDVi-FITC for 40 or 120 min at 37°C or at 4°C cells were then analyzed by flow cytometry before and after the addition of Trypan Blue (0.01 mg/ml). Fluorescence was measured in a BD FACSCalibur flow cytometer (BD Biosciences, CA, USA) and the data was analyzed using FlowJo software (FlowJo).

### 2.6 EVs isolation

Small EVs derived from APC were isolated by differential centrifugation, ultrafiltration, and ultracentrifugation. Briefly, conditioned medium of APC pulsed with FMDVi (EVs-FMDVi), from untreated APC that were incubated only with RPMI (EVs-RPMI), and from LPS-activated APC (EVs-LPS) was centrifuged at 300x*g* for 10 min to separate floating cells. Supernatants were filtered through a 0.22 μm porous membrane and ultracentrifuged at 100,000x*g* for 80 min. at 4°C. The pellet with EVs were resuspended in phosphate-buffered saline (PBS) and were concentrated in the last step of 70 min ultracentrifugation at 100,000x*g*. The pellet obtained was resuspended in PBS and EVs were characterized by flow cytometry after each isolation and stored at -80°C. To standardize the number of vesicles, all EVs derived from a total of 10.00 x 10^6^ APC were resuspended in a volume of 50 μl of PBS (20). Only cultures with viability more than 85% as assessed by Trypan blue exclusion staining were used for EV isolation. Viability measurement was consistently performed after each pulsing experiment and before and after each vesicle purification experiment.

### 2.7 EVs immunofluorescence staining and flow cytometry

EVs derived from 5.00 x 10^6^ APC (25 μl) were incubated with 10 μl of latex beads aldehyde/sulfate (Invitrogen^®^/Thermofisher, CA, USA 4% W/V- 4-μm diameter). After being saturated with 100 mM glycine, EVs were incubated with the following monoclonal antibodies: 0.02µg/100µl anti-MHC-II-PE (Biolegend-clone: M5/114.15.2), 0.2µg/100µl anti-CD11c-APC (Biolegend-clone: N418), 1.5 µg/100µl anti-CD86-FITC (Miltenyi-clone: PO3.3), 1 µg/100µl anti-CD9-biotin (eBioscience-clone: MEM61), 1 µg/100µl anti-CD81-biotin (eBioscience clone: Eat2) or 1 µg/100µl polyclonal antibody anti-CD63-biotin (MyBioSource). After incubation with the primary antibody, the cells were washed and then incubated with 0,04 µg/100µl streptavidin-phycoerythrin (PE) conjugated (eBioscience). Analysis was performed on the singlet gate of a forward scatter versus a side scatter dot plot (21). Fluorescence was measured in a BD FACSCalibur flow cytometer (BD Biosciences, CA, USA). The data were analyzed with FlowJo X software.

### 2.8 Size distribution by nanoparticle tracking analysis

The diameter size of the vesicle population was determined using equipment ZetaView PMX‐120 (Particle Metrix). The samples were evaluated using various dilutions. The software analyzed raw data videos in triplicate (22).

### 2.9 Expression of viral protein on EVs at different secretion and pulse times

EVs-beads were incubated with anti-FMDV-FITC (IgG purified from the serum of immunized bovine). IgG purified from the bovine serum of a healthy animal was used as IgG control. Analysis was performed on the singlet gate of a forward scatter versus a side scatter dot plot. Fluorescence was measured in a BD FACSCalibur flow cytometer (BD Biosciences, CA, USA). The data were analyzed using FlowJo software (FlowJo).

### 2.10 Carboxyfluorescein succinimidyl ester proliferation assay

BALB/c mice were immunized with monovalent FMDV O1 Campos vaccines i.p. On day 14, spleen cells were obtained under sterile conditions. The proliferation of lymphocytes was evaluated by the CFSE dilution. The CFSE staining was performed using the CFSE (Invitrogen, CA, USA) at a final concentration of 5 μM for 10 min at room temperature, followed by immediate quenching with culture medium. CFSE-stained splenocytes (3.00 × 10^5^/ml) were seeded in a 96-well plate and incubated for 5 days with EVs-FMDVi or EVs-LPS derived from 2.50 x 10^6^ APCs, or 10 μg/ml of FMDVi. For the positive and negative controls, splenocytes were incubated with 0.5 μg of Con A and RPMI complete medium, respectively. The antigen-specific proliferation of cells was determined by incubation with purified FMDVi. To analyze T cell subpopulations, stimulated and CFSE-stained splenocytes were incubated with PE-conjugated anti-CD3 or anti-B220 monoclonal antibodies. Anti-CD21 APC and anti-CD23 Biotin antibodies were also used for the study of B cell subpopulations. Streptoavidin-PECy5 was used as a conjugate. For all *in vitro* experiments, the EVs were previously sterilized by filtration using a 0.22 μm filter. *In vitro* stimulated splenocytes were analyzed by flow cytometry. Fluorescence was measured in a BD FACSCalibur flow cytometer (BD Biosciences, CA, USA). The data analysis was performed using FlowJo software (FlowJo). Relative proliferative index (RPI) is defined as the ratio between the percentages of stimulated cells and the percentage of control cells.

### 2.11 Statistical analysis

The statistical significance of differences between the experimental and control groups was analyzed using one-way ANOVA followed by a post-ANOVA Tukey’s multiple comparison test or Bonferroni multiple comparisons test. For certain analyses, the non-parametric Kruskal-Wallis method was used followed by the Dunn multiple comparisons test to compare pairs of groups. All analyses used are detailed below each figure in the results section. Differences were considered significant at p values < 0.05 for all comparisons. The statistical analysis was performed using GraphPad Prism software 8 for Windows (GraphPad Software, San Diego, California, USA).

## 3 Results

### 3.1 APC internalize the FMDVi O1 Campos vaccine antigen and become activated *in vitro*

#### 3.1.1 Virus internalization by APC promotes its activation

**Figure 1:**
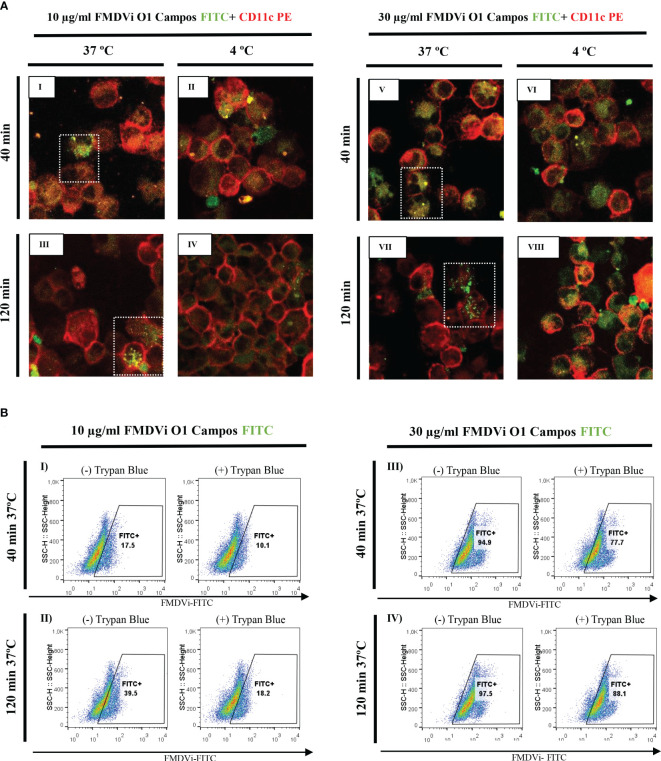
FMDVi internalization by APC. **(A)** Confocal microscopy of APC incubated at 37°C or 4 °C with I-IV) 10 µg/ml FMDVi-FITC; V-VIII) 30 µg/ml FMDVi-FITC. The images show the expression of the CD11c marker (red) on the cell membrane and FMDV labeled with FITC (green) inside the cell membrane. Highlighted with a dotted line square are the cells that internalized FMDV-FITC. A representative field of 5 fields captured for each experimental condition is shown in the image. **(B)** Flow cytometry of APC incubated with I and II) 10 µg/ml FMDVi-FITC; III and IV) 30 µg/ml FMDVi-FITC. The shift to the right of the population can be observed, showing an increase in the fluorescence intensity corresponding to FITC. The analysis was performed in the region corresponding to APC according to SSC and FSC parameters. The graphs show the different incubation times and the presence or absence of Trypan Blue dye.

To elucidate the interaction between APC and the antigen used for commercial FMDV vaccine (BEI-FMDVi), we differentiated APC from murine bone marrow pluripotent precursors. Differentiated APC were obtained after 8 days of culture with GM-CSF as detailed in material and methods. The interaction between APC with FMDVi was assessed by confocal microscopy and flow cytometry (**Figures 1A, B**, respectively). APC cells were incubated with different concentrations (10 and 30 µg/ml) of FMDVi conjugated with the fluorochrome FITC (FMDVi-FITC). FMDV internalization was measured at 40 and 120 min. At both times and both virus concentrations, by confocal microscopy, we observed that APC (CD11c+) can internalize the FMDVi-FITC when incubating at 37°C (**Figure 1A I, III, V, and VII** square in dotted line). Upon repeating the incubation at 4°C, the internalization process slows down due to the decrease in the temperature (**Figure 1A II, IV, VI, and VIII**). These results were confirmed by flow cytometry where virus internalization was reflected as a fluorescence shift to the right (**Figure 1B I-IV**). In the group treated with 10 μg/ml FMDVi-FITC, the population FITC+ was 17.5% at 40 min (**Figure 1B I**) and increased to 39.5% at 120 min (**Figure 1B II**). When 30 ug/ml of the virus was used, it was observed that most of the virus was internalized at 40 min (94.9%) and 120 min (97.5%) (**Figure 1B III and IV**, respectively). After the addition of Trypan Blue, the fluorescence decreased as a result of the quenching effect allowing discriminating internalized virus microparticules from attached to the cell membrane. In the group corresponding to 10 μg/ml FMDVi-FITC and 40 min incubation, the FITC-positive population decreased a 7.4% (**Figure 1B I**). On the contrary, in the group corresponding to 30 μg/ml of FMDVi-FITC and 40 min, the decrease was 17.2% (**Figure 1B III**). When evaluating both concentrations (10 and 30 μg/ml of FMDVi-FITC) but at 120 min incubation time, a 21.3% decrease in fluorescence was observed for the 10 μg/ml group and 9.4% for the 30 μg/ml group (**Figure 1B II and IV**). These results indicate that murine APC interact and internalize the FMDVi vaccine antigen. The internalization of FMDVi by APC is supported by the presence of FITC+ particles inside the cell and the fluorescence persistence after the addition of Trypan Blue.

**Figure 2:**
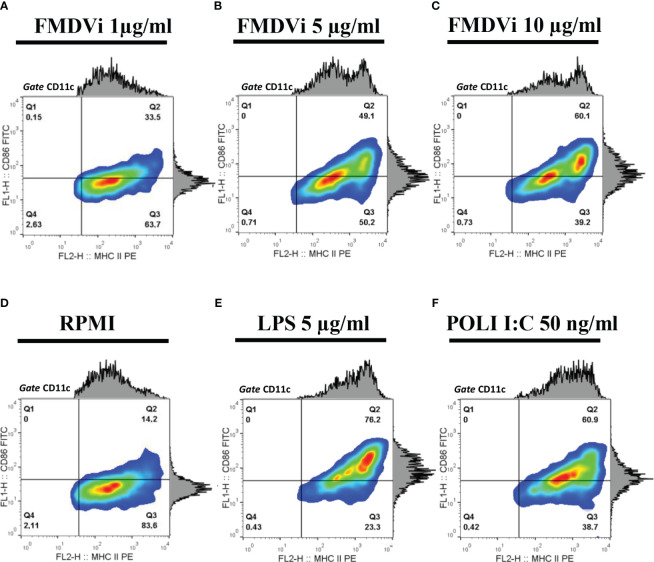
Activation of APC by FMDVi. APC were incubated with: **(A)** 1 μg/ml of FMDVi; **(B)** 5 μg/ml of FMDVi or **(C)** 10 μg/ml of FMDVi for 16 h at 37°C in 6-well plates. **(D)** Unstimulated cells (RPMI) were used as a negative control. **(E)** Cells cultured for 16 h with 5 μg/ml LPS or **(F)** 50 ng/ml POLI I: C were used as positive controls. For the analysis, a *gate* was performed according to the data obtained from SSC vs FSC. MHCII + population was then defined within the CD11c+/MHCII+ cell region. Contour density plots and histograms corresponding to CD86 and MHC-II expression are shown.

To evaluate the activation of APC after FMDVi internalization we incubated the APC with increasing concentrations of FMDVi, and then the expression of the activation markers MHC-II and CD86 were assessed by flow cytometry (**Figure 2**). CD86 and MHC-II expression in untreated APC (negative control), LPS or POLI I:C treated APC (positive controls) was shown in **Figures 2D–F**, respectively. As can be seen in **Figures 2A–C**, APC activation progressively increases with virus concentration. This is reflected as an increase in the percentage of MHC-II+/CD86+ cells. This result was consistent with the phenotype of activated DC. This experiment revealed that inactivated viral particles can activate APC by inducing the upregulation of MHC-II and CD86 molecules necessary for the generation of an adaptive response. Based on these results, the working concentration of FMDVi for optimal stimulation of APC was 10 µg/ml, as the highest percentage of activated MHC-II^+^/CD86^+^ cells was achieved without altering the cell viability of the culture.

### 3.2 Bone marrow-derived APC loaded with FMDVi secrete EVs expressing viral proteins

#### 3.2.1 APC release EVs expressing viral proteins only during the first 24 h post-internalization

**Figure 3:**
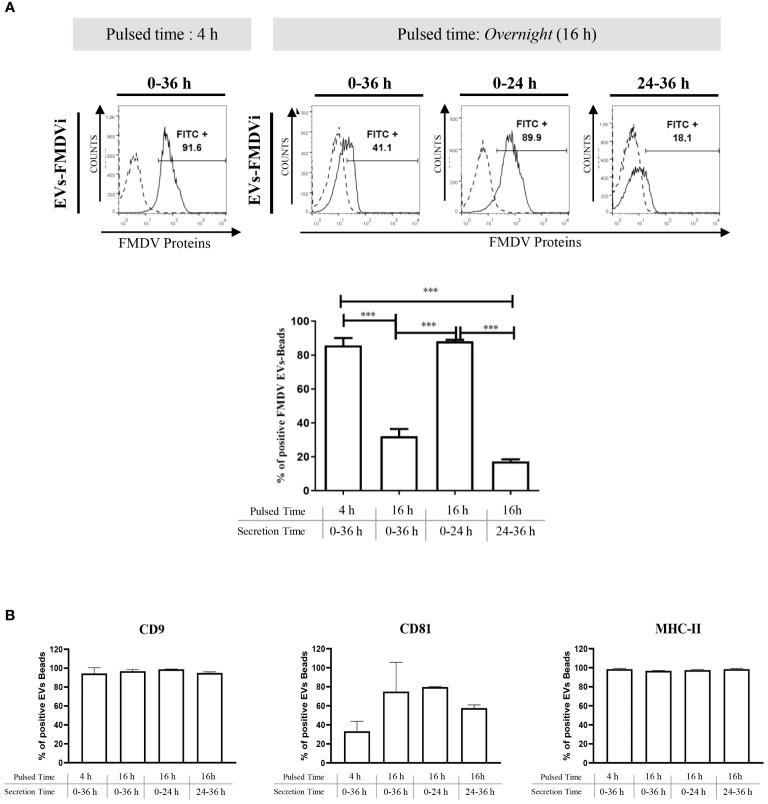
FMDVi-activated APC secretes EVs expressing viral proteins. **(A)** EVs were coupled with aldehyde/sulfate latex beads of 4 µm diameter. For viral protein detection on EVs, FITC-labeled IgG purified from an FMDV-immune bovine serum was used, and FITC-labeled IgG obtained from a healthy unimmunized bovine serum was used as a corresponding control (Control). Flow cytometry histograms corresponding to the expression of viral proteins in EVs at different pulsed times. Analysis was performed on the *singlet gate* of the SSC vs FSC plot. Histograms with continuous lines represent EVs–bead complexes stained with specific monoclonal antibodies, and histograms with dotted lines represent bead’s autofluorescence control. Columns bar graph details the pulsing time and the times at which supernatants were collected and EVs were purified (secretion time). **(B)** To assess the secretion of EVs at each time point EVs were incubated with anti-CD9 and anti-CD81. The origin from APC was determined by the expression of MHC-II molecules. Data represent the mean value ± SEM of 4 independent experiments. One-factor ANOVA was used for statistical analysis, followed by comparisons using a post-ANOVA Tukey’s multiple comparisons test. Asterisks indicate significant differences (***p < 0.001).

To evaluate the role of EVs in the antiviral immune response, EVs from the conditioned medium of FMDVi-pulsed APC were isolated. The optimal time to harvest EVs was determined by studying the expression of FMDV proteins. Two pulse times (4 h and 16 h) and three secretion times (24 h, 36 h, and a third time divided into two intervals: the initial 24 h and the final 12 h covering a total of 36 h) were analyzed (**Figure 3**). The results revealed that there was a significant increase in the secretion of EVs expressing FMDVi proteins (CD9+/CD81+/FMDVi+) during the first 24 h after pulsing APC with FMDVi for 16 h (p<0.001 when compared to the other secreted time points, 0-36 h and 24-36 h) (**Figure 3A**). Afterward, APC continued secreting vesicles for the next 12 h but lacked viral antigens (CD9+/CD81+/FMDVi-) (**Figure 3A**). Comparable viral protein expression was achieved by shortening the pulse time to 4 h and extending the secretion of EVs to 36 h (**Figure 3A**). The presence of EVs was confirmed at all evaluated time points through the expression of CD9 and CD81 proteins (**Figure 3B**). Furthermore, their cellular origin from APC was assessed through MHC-II expression (**Figure 3B**).

#### 3.2.2 Characterization of EVs derived from APC according to size, morphology, and protein profile

EVs isolated from a conditioned medium of APC pulsed with FMDVi (EVs-FMDVi), from untreated APC that were incubated only with RPMI (EVs-RPMI), and from LPS-activated APC (EVs-LPS) were biophysically characterized by electron microscopy (TEM), nanoparticle tracking analysis (NTA) and flow cytometry, following the guidelines given by ISEV in the *minimal information for studies of extracellular vesicles* (MISEV) (23).

**Figure 4:**
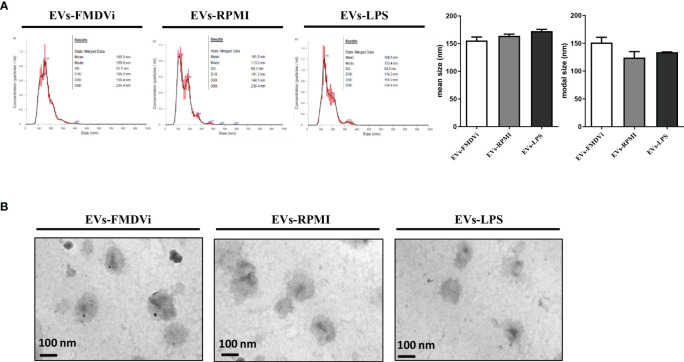
The secreted EVs-FMDVi are classified as small EVs, based on their size and morphology. **(A)** The size distribution was evaluated from nanoparticle tracking analysis. Representative NTA profiles of EVs isolated from APC loaded with FMDVi antigen (EVs-FMDVi), unstimulated APC (EVs-RPMI), or from APC activated with LPS (EVs-LPS). Plots correspond to one measurement made for each treatment in a total of 4 independent measurements. Column bar graph represents the mean or modal values ± SEM. The Kruskal Wallis test was used for statistical analysis, followed by a Dunn’s multiple comparisons test. **(B)** Electron micrograph of EVs isolated from APC conditionate medium of each treatment. The size (100-200 nm) and typical cup shape of the EVs can be observed. Nine images of each sample were obtained at 50,000X magnifications.

NTA analysis showed that all the EV populations obtained had a relatively homogeneous and similar size distribution, with no differences between them (**Figure 4A**). The average size for EVs-FMDVi was 155.7 nm ± 6.25, 164.1 nm ± 3 for EVs-RPMI, and 172.7 nm ±3.15 for EVs-LPS. The sizes of the EVs obtained from APC correspond to those reported for nanovesicles, particularly for small EVs (sEVs). TEM micrograph (**Figure 4B**) shows that the EVs obtained from the different treatments presented the cup-shaped or flat balloon shape characteristic for exosomes (24). It was also possible to confirm their size (around 150 nm) which agrees with the values reported in the literature for sEVs and in concordance with the size obtained from NTA analysis. In agreement with previous results, EVs morphology was also not affected by the activation treatments to which the APC were exposed.

**Figure 5:**
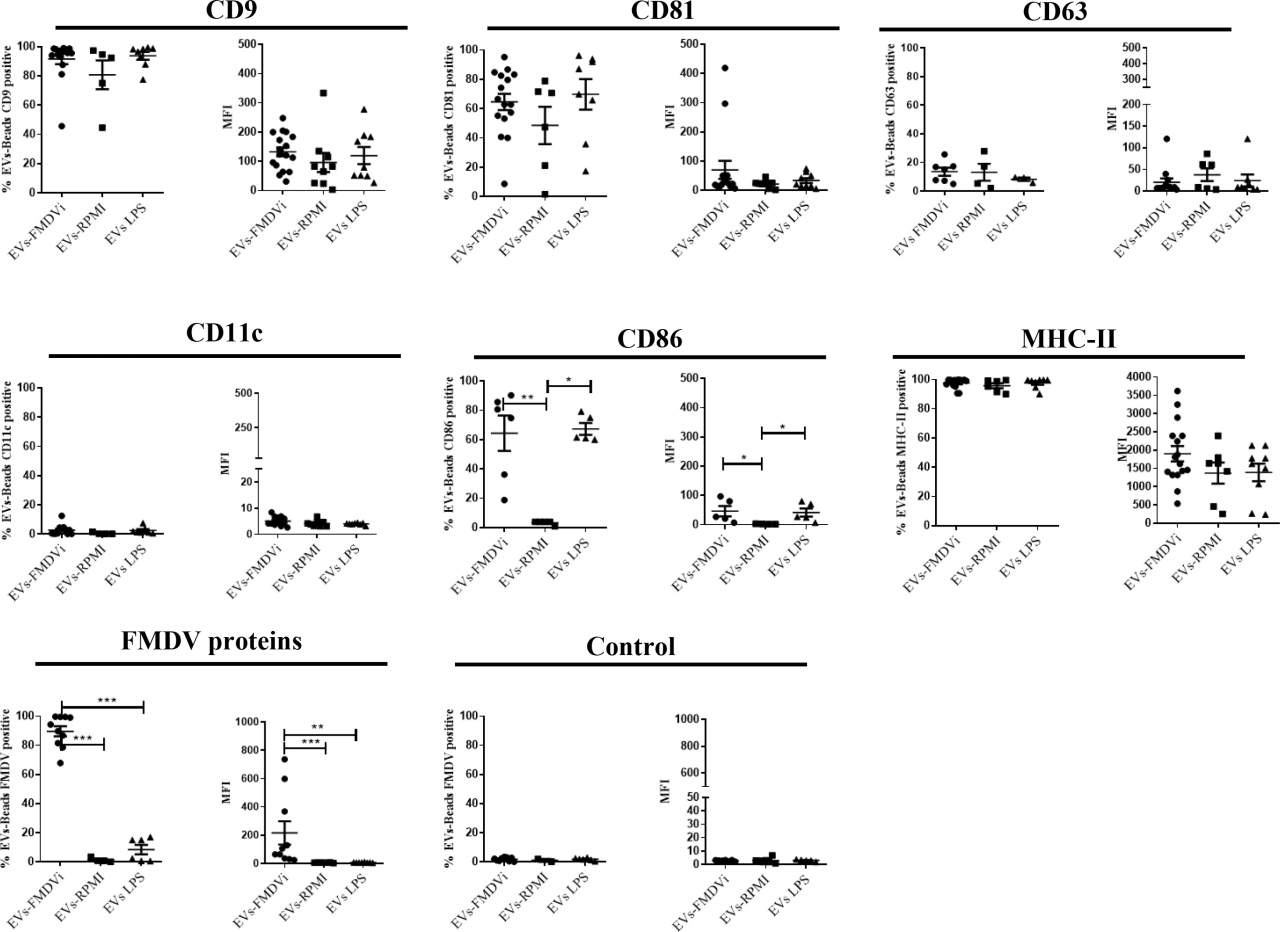
EVs-FMDVi express FMDV proteins, classical EVs markers, and immunoregulatory molecules. EVs were coupled with aldehyde/sulfate latex beads of 4 µm diameter and incubated with different fluorochrome-conjugated monoclonal antibodies anti-CD9, anti-CD81, CD63, MHC-II, CD11c, CD86 as detailed in materials and methods. For viral protein detection on EVs, FITC-labeled IgG purified from an FMDV-immune bovine serum was used, and FITC-labeled IgG obtained from a healthy unimmunized bovine serum was used as a corresponding control (Control). Comparison of percentages for different marker proteins and FMDV viral proteins for each EV population. The mean value ± SEM of the Median Fluorescence Intensity (MFI) for each of the markers evaluated is also shown. For statistical analysis, the Kruskal-Wallis test followed by Dunn’s multiple comparisons test was used for the analysis of MFI, and one-factor ANOVA followed by Bonferroni’s multiple comparisons test for the analysis of percentages. Only significant differences are indicated in the graphs (p<0.05 *; p<0.01**; p<0.001 ***). The data correspond to 15 independent experiments.

As shown in **Figure 5** and **Supplementary Figure S2**, EVs derived from different treatments expressed the tetraspanins marker CD9, CD81, and CD63. CD9 is the most expressed protein, being present in roughly 90% of EVs-FMDVi or EVs-LPS and nearly 80% in EVs-RPMI (**Figure 5**). All EV populations obtained expressed a high percentage of MHC-II molecule, being present in more than 90% of each population (**Figure 5**). Surprisingly, CD11c marker, although present in abundance in the DC, was not detected in any of the EV populations obtained. Significant differences were found when analyzing CD86 (**Figure 5**). EVs derived from activated APC expressed a higher level of CD86 than EVs-RPMI, reflected in both, the percentages of EVs expressing CD86 (64.42% ± 12.07 for EVs-FMDVi, 67.36% ± 4.06 for EVs-LPS, and 3.27% ± 0.57 for EVs-RPMI) and in the MFI (MFI: 46.4 ± 17.7 for EVs-FMDVi, MFI: 41.71 ± 14.11 for EVs-LPS, and MFI: 2.41± 0.25 for EVs-RPMI). These findings suggest that the expression of CD86 in EVs would reflect the activation status of the APC from which they come. On the other hand, only EVs derived from APC pulsed with FMDVi expressed viral antigens in more than 85% of the population (p<0.001 when comparing EVs-FMDVi with, EVs-RPMI or EVs-LPS) (**Figure 5**) reflected as well as in MFI (p<0.001 and p<0.01 when comparing FMDVi-EVs with RPMI-EVs and LPS-EVs, respectively).

It is important to note that the possibility that the viral proteins expressed in EVs-FMDVi could be derived from viral debris copurified with the EVs was excluded by an additional experimental control. The control consisted of performing the same incubation protocol with FMDVi but without APC. Then the supernatant obtained was processed following the EV isolation protocol and characterized by flow cytometry. As expected, neither EVs markers CD9, CD81, CD63 nor FMDV antigens were found. The absence of viral proteins indicates that no viral remnants, which could co-purify with the EVs, were isolated (data not shown).

The data obtained from the characterization demonstrated that the treatment of APC with FMDVi or LPS does not affect the size or expression of protein markers in the EVs. The principal differentiating characteristics between the various EV groups are the presence or absence of viral proteins (only expressed in EVs-FMDVi) and the absence of CD86 in those EVs derived from unstimulated APC (EVs-RPMI). Our results confirm that EVs from different treatments have similar characteristics, and only differ in the expression of FMDVi antigens and CD86.

### 3.3 EVs-FMDVi induce an antigen-specific lymphocyte proliferation *in vitro*

To assess whether viral proteins expressed by EVs-FMDVi could be recognized by lymphocytes previously primed with the FMDVi *in vivo*, an antigen-specific lymphoproliferation assay was performed using the CFSE dye dilution technique. Splenocytes obtained from mice immunized with FMDVi O1 Campos (IMMUNE) as well as those obtained from naïve mice (NAÏVE) were stained with CFSE and stimulated *in vitro* with EVs-FMDVi, FMDVi or Con A as a positive control. EVs-LPS were used as a control for stimulated APC-derived EVs that do not express viral proteins.

**Figure 6:**
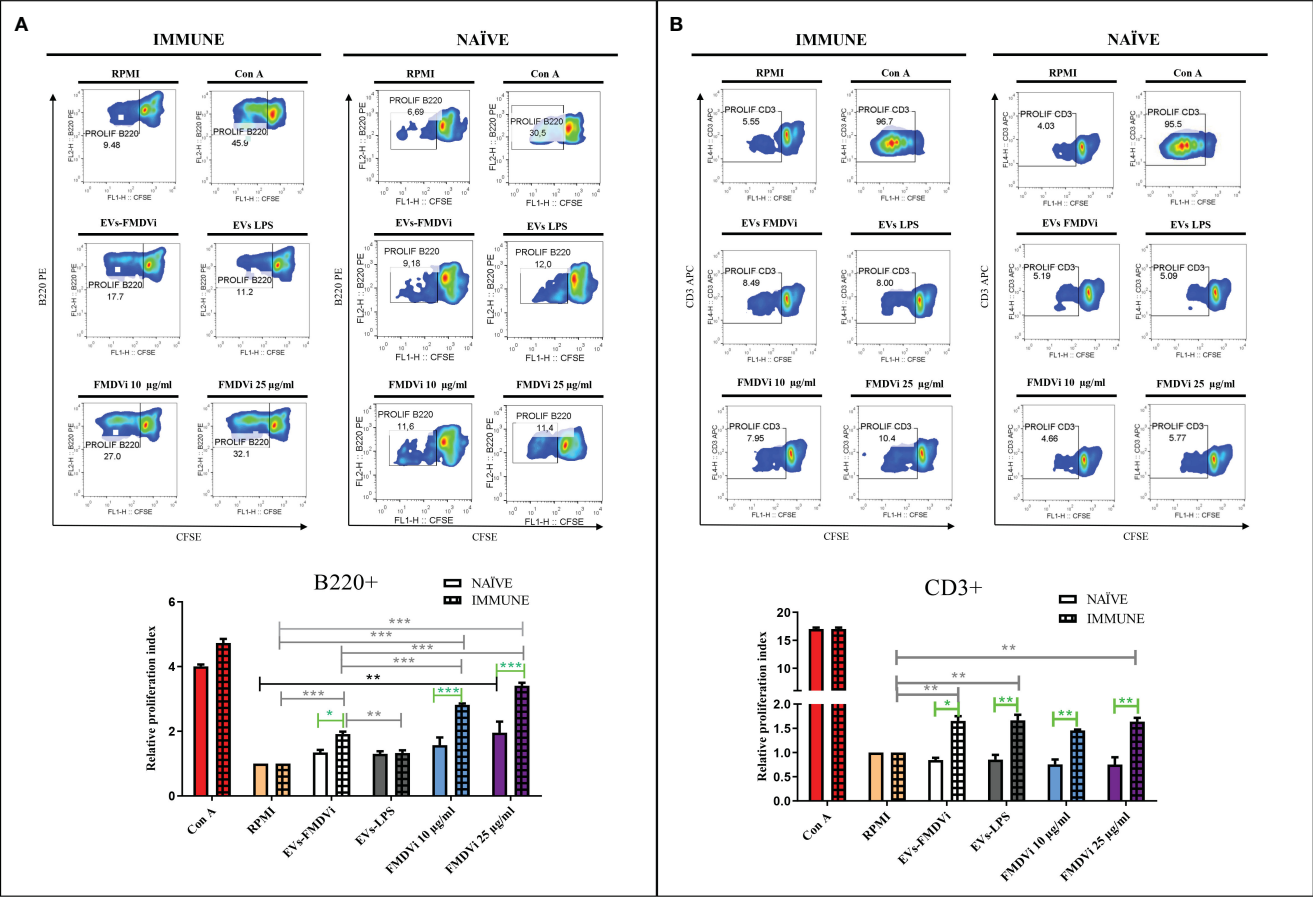
EVs-FMDVi induces antigen-specific lymphoproliferation. Lymphoproliferative response of **(A)** B220+ lymphocytes stimulated with EVs-FMDVi, **(B)** CD3+ lymphocytes stimulated with EVs-FMDVi. Splenocytes obtained from mice primed *in vivo* with the monovalent FMDVi O1 Campos vaccine (INMUNE) were labeled with 5 μM CFSE and cultured for 5 days at 37°C with EVs-FMDVi or EVs-LPS isolated from 2.50 x 10^6^ APC, 10 or 25 µg/ml FMDVi. Unstimulated splenocytes (RPMI) stained with CFSE were used as a negative control and splenocytes stimulated with 0.50 μg/ml Con A were used as a positive control for nonspecific stimulation. The same protocol was used for splenocytes obtained from naïve mice (NAÏVE). Flow cytometry density plots correspond to one representative of three independent experiments. The measurements were performed in triplicate. The lymphocyte region was determined from the SSC vs FSC plot. Proliferation was analyzed in the region corresponding to B220+ or CD3+ lymphocytes. Bars represent the mean value ± SEM of the relative proliferation index (RPI), defined as the ratio between the percentages of stimulated cells by the percentage of basal proliferation (RPMI). For statistical analysis, a one-factor ANOVA was performed, followed by a Bonferroni multiple comparisons test. Two-factor ANOVA was used for comparisons between NAÏVE vs IMMUNE. Only significant differences are indicated in the graphs which are marked with asterisks (p<0.05*; p<0.01**; p<0.001***). Significant differences for comparisons within the IMMUNE group are shown in gray line, significant differences for comparisons within the NAÏVE group are shown in black line, and significant differences for comparisons between NAÏVE vs IMMUNE are shown in green line. Solid bars represent naïve mice and grid bars represent immunized mice. N=6.

To determine which cell subpopulations were stimulated by the presence of the viral antigens expressed on the EVs, T and B cell populations were analyzed (**Figure 6**). Proliferation assays showed that sensitized B220+ cells (IMMUNE) proliferate in an antigen-specific manner in the presence of EVs-FMDVi (**Figure 6A** left panel). This proliferation was significantly higher when compared to basal proliferation (RPMI) or to non-specific stimulated splenocytes incubated with EVs-LPS (p<0.001 and p<0.01, respectively) (**Figure 6A** column bar graph, gray line). Although EVs-FMDVi stimulated B cell proliferation, this proliferation was approximately 30% lower compared to those stimulated by 10 μg/ml FMDVi vaccine antigen (mean RPI: 1.91 and mean RPI: 2.8, respectively) and roughly 40% lower compared to 25 μg/ml FMDVi (mean RPI: 3.4). On the other hand, the same assay using naïve lymphocytes (**Figure 6A** right panel) showed that only the concentration of 25 μg/ml FMDVi induced a statistically significant proliferation of B220+ cells respect to the basal control (p<0.01, mean RPI: 1.95. **Figure 6A** column bar graph, black line). When comparing NAÏVE vs IMMUNE group, a higher proliferation was observed in the IMMUNE group for all the treatments (**Figure 6A**, column bar graph, green line). This proliferation was higher for both EVs-FMDVi treatment (EVs-FMDVi NAÏVE vs EVs-FMDVi IMMUNE, p<0.05), and for both FMDVi concentrations evaluated (10μg/ml FMDVi NAÏVE vs 10μg/ml FMDVi IMMUNE, p<0.001; 25 μg/ml FMDVi NAÏVE vs 25μg/ml FMDVi IMMUNE, p<0.001. **Figure 6A**, column bar graph, green line). However, for the treatment corresponding to EVs-LPS, no stimulation was observed in NAÏVE or IMMUNE B lymphocytes. Also, T cell stimulation was observed when incubated with EVs-FMDVi. **Figure 6B** shows a significant proliferation in the group of CD3+ lymphocytes primed *in vivo* with FMDVi (p<0.01, compared to baseline; mean RPI: 1.65. **Figure 6B**, column bar graph, gray line). However, similar lymphoproliferation was observed when lymphocytes were incubated with EVs-LPS (p<0.01; mean RPI: 1.66. **Figure 6B**, column bar graph, gray line). Therefore, we cannot state that the proliferation induced by EVs-FMDVi is antigen-specific. Furthermore, EVs or FMDVi did not stimulate naïve splenocytes demonstrating that both EVs-FMDVi and EVs-LPS induced proliferation only in T cells that had been previously primed *in vivo* with the virus. It was found that both EVs-FMDVi and EVs-LPS induced proliferation only in T cells that had been previously primed *in vivo* with FMDVi, indicating that prior exposure to the virus was necessary for T cell activation.

**Figure 7:**
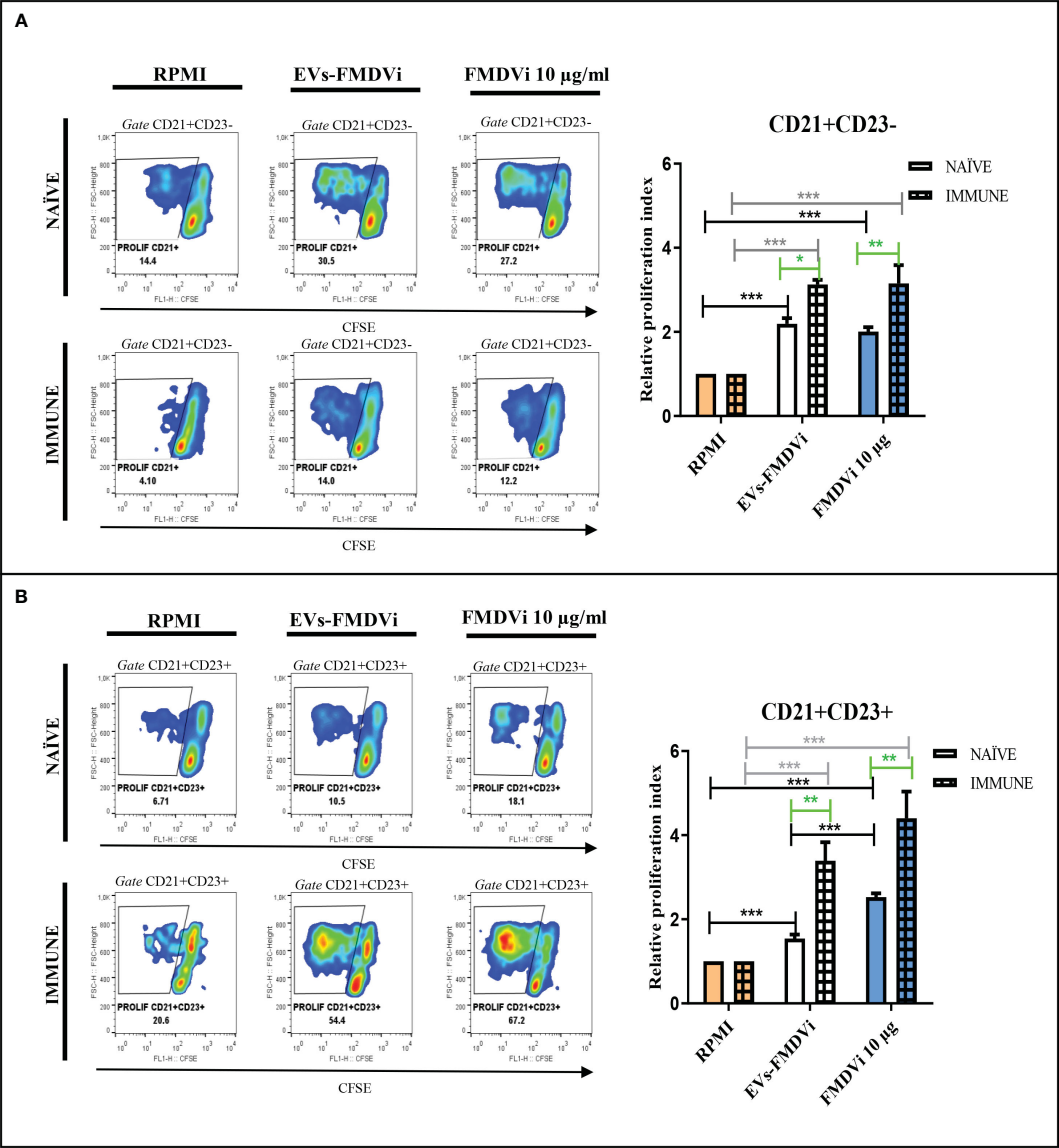
EVs-FMDVi induces virus-specific lymphoproliferation in B cell subpopulations. Lymphoproliferative response of **(A)** CD21+/CD23- lymphocytes stimulated with EVs-FMDVi, **(B)** CD21+/CD23+ lymphocytes stimulated with EVs-FMDVi. Splenocytes obtained from naïve mice (NAÏVE) or immunized with the monovalent FMDVi O1 Campos vaccine (IMMUNE) were stained with CFSE and cultured for 5 days at 37°C with EVs-FMDVi isolated from 2.50 x 10^6^ APC and 10µg/ml FMDVi. Unstimulated CFSE-stained splenocytes (RPMI) were used as a negative control. The graphs correspond to one experiment, measurements were performed in quintuplicate. The lymphocyte region was determined from the SSC vs FSC graph. Proliferation was analyzed in the region corresponding to CD21+/CD23- or CD21+/CD23+ lymphocytes. A two-way ANOVA was performed, and pairwise comparisons were conducted using a post-ANOVA Bonferroni multiple comparison test. Only significant differences are indicated in the graphs marked with asterisks (p<0.05*; p<0.01**; p<0.001***). Significant differences for comparisons within the IMMUNE group are shown in gray line, significant differences for comparisons within the NAÏVE group are shown in black line, and significant differences for comparisons between NAÏVE and IMMUNE are shown in green line. The relative proliferation index (RPI) was defined as the ratio of the percentage proliferation of the experimental group over the percentage basal proliferation (RPMI). Bars represent the mean ± SEM percentage of relative proliferation index (RPI). Solid bars represent naïve mice and grid bars represent immunized mice. (N=5).

Knowing that EVs-FMDVi induce antigen-specific proliferation in B cells and the importance of the B cell response in FMDV infection, different subpopulations of B cells were evaluated. Spleen marginal zone lymphocytes (MZB) are characterized by high expression of CD21+ and low or no expression of the CD23 marker, while the follicular lymphocytes (FoB) express both CD21 and CD23 (25). EVs-FMDVi were used as an antigen-specific stimulus and the vaccine antigen FMDVi 10 μg/ml was used as a positive control. Both, EVs-FMDVi and FMDVi induced a significant increase in the proliferative response of CD21+/CD23- lymphocytes when compared to unstimulated control in IMMUNE and NAÏVE group (p<0.001 for both IMMUNE and NAÏVE splenocytes. **Figure 7A**, column bar graph black line and gray line, respectively). However, RPI in IMMUNE was always higher than in NAÏVE splenocytes (43% higher for EVs-FMDVi and 57.5% higher for FMDVi) (**Figure 7A**, column bar graph). This implies that EVs-FMDVi can stimulate the proliferation of MZB (CD21+/CD23-) cells *in vitro* as FMDVi antigens (**Figure 7A**, column bar graph).

As observed for CD21+/CD23- lymphocytes, both the EVs-FMDVi and the FMDVi consistently induced and increased CD21+/CD23+ lymphocyte proliferation compared to the basal level (RPMI). This was observed in both the NAÏVE group and the IMMUNE group (EVs-FMDVi p<0.001 and FMDVi p<0.001. **Figure 7B**, column bar graph, black line and gray line). An enhanced cellular expansion was observed in CD21+/CD23+ lymphocytes that were previously sensitized *in vivo* with FMDVi (p<0.01. **Figure 7B**, green line). The RPI was increased by 120% and 74% (for EVs-FMDVi and FMDVi, respectively), compared to naïve splenocytes.

All these data showed that proteins/peptides expressed on the EVs-FMDVi can activate both CD21+/CD23- and CD21+/CD23+ cells, from both previously sensitized with FMDVi and naïve lymphocytes.

## 4 Discussion

Communication plays a leading role in the immune system, cell-cell interactions and soluble factors are fundamental. A few years ago, EVs were incorporated as another means of communication between cells and can activate or inhibit an immune response depending on the originating cell’s state (3). For example, EVs derived from DCs reflect the activation and functional state of the parent cell and have an active role in the immune response. Previous studies have shown that FMDV can affect murine DCs differently depending on whether it is infectious or inactivated. (26, 27). Based on these considerations, we hypothesize that EVs derived from APC exposed to FMDV O1 Campos (an antigen commonly found in commercial vaccines) may activate the immune response to FMDV. This activation could be a significant factor in the development of the protective immunity conferred by the vaccine.

EVs were isolated from culture-conditioned supernatant of APC pulsed with FMDVi, LPS, or untreated APC, using centrifugation, ultrafiltration, and ultracentrifugation. The isolated EVs-FMDVi, EVs-RPMI, and EVs-LPS were similar in shape and size, ranging from 100-200 nm, and enriched in sEVs or exosomes. These EVs expressed high levels of tetraspanin CD9, moderate levels of CD81, and low levels of CD63. In addition, they also express immunomodulatory molecules, such as the MHC-II, and the costimulatory molecule CD86 indicating that they arise from APC. Furthermore, the fact that CD86 was higher in EVs derived from mature APC such as those pulsed with FMDV or activated with LPS demonstrates that EVs reflect the maturation state of the cells of origin. Since the CD11c molecule is widely used to characterize bone marrow derived DCs, we included it in the characterization. Surprisingly, although abundant in DCs, this protein was not detected in the EVs populations studied. In line with our findings, only a few papers from the extensive bibliography consulted, report the expression of CD11c in EVs derived from DCs, and its presence is only detected when specific isolation methodologies were used (28). In conclusion, from our results and following MISEV guidelines, we defined the EVs derived from APC we isolated as a small population of EVs with high expression of the tetraspanin CD9, medium expression of CD81, and low expression of CD63 (1).

It is well known that during its maturation, DCs acquire proteins from pathogens or microorganisms that are later processed for T cell presentation. Considering this, EVs obtained from FMDVi-activated APC could have associated FMDVi proteins or peptides. Our experiments demonstrated that FMDVi proteins/peptides were detected in EVs-FMDVi. These viral proteins/peptides could be either bound to the MHC, associated with the EVs surface, or included in the membrane of the EVs. Although we did not perform any experiments to determine the location of these proteins, we can infer from the flow cytometry results that they are expressed on the surface of the EVs, since they could interact with FMDV-specific antibodies.

As mentioned above, DCs secrete in their external environment different amounts and types of EVs according to the microenvironment and stimuli (29). Changes in the cell of origin may impact the number of EVs secreted and their phenotype. Furthermore, the composition of EVs subpopulations isolated at a one-time point is determined by the balance between the EVs secreted by a cell and those that are reincorporated into an accepting cell (30).Considering these facts and knowing that antigen persistence in DCs is influenced by several factors, including degradation rate, cell protein recycling, and antigen availability in the external environment, we isolated EVs-FMDVi from DCs pulsed with FMDVi at different incubation-secretion times. DCs pulsed with FMDVi do not secrete EVs-FMDVi constantly instead have a limited secretion period. Within the initial 24 h after pulsing, DCs secrete EVs containing viral proteins. Beyond this time frame, these DCs continue to release EVs with identical characteristics in EV markers (CD9 and CD81) and immunocompetent molecules (MHC-II) but do not express viral proteins. One probable explanation for this unexpected result could be that all the antigens internalized in the APCs have already been processed within 24 h and therefore the EVs no longer express viral proteins. Collecting EVs-FMDVi beyond 24 h may result in a dilution effect for those EVs expressing viral proteins.

FMDVi antigens are present on EVs membranes, either unprocessed or partially processed since EVs-FMDVi induce specific B cell proliferation, and also because antibodies present in sera of immunized animals with the commercial FMDV vaccine (used in the protein characterization by flow cytometry) can recognize EVs-FMDVi. This is important because B cell epitopes are often discontinuous and many FMDV antigenic sites are conformational. For example, the G-H loop of the VP1 protein is a crucial conformational antigenic site (31). Previous studies have demonstrated that proteins expressed on EVs induce humoral immune responses both *in vitro* and *in vivo* (32). Our findings are also in line with this research and the one conducted by Gabrielson’s team, which first demonstrated that EVs derived from pulsed DCs activate specific immune cells (33). Furthermore, our results and those of previous studies suggest that antigens recognized and endocytosed by APC can be redirected intact to the membrane of EVs. According to our research, Dr. Zheng’s group has shown that, in infective FMDV, all FMDV proteins (excluding proteins L, 2A, and 3B) were included in exosomes that were purified from the infected PHK-15 cells supernatant. Additionally, the vesicles contained the entire genomic RNA of the virus (34). These findings and our results suggest that exosomes and EVs play a crucial role in transporting FMDV viral materials.

The role of EVs derived from DCs in antigenic presentation to T cells is well known. However, little is known about their role in the activation of B cells and the generation of a humoral response (35). We mainly focused the study on marginal MZB (CD21+/CD23-) and FoB (CD21+/CD23+) because they are involved in FMDVi immune response. MZB lymphocytes play an important role in the germinal center by transporting antigens from the blood to the follicular DCs (FDCs) in the spleen (36, 37) and exhibit the kinetics and activation requirements typical of the innate system (38–41). The MZB lymphocytes are responsible for generating the first FMDVi-neutralizing antibodies. On the other hand, FoB cells which constitute the predominant population of mature B cells participate in the production of high-affinity antibodies and in the generation of memory B cells that will protect from reinfections (42). Through Ag-specific lymphoproliferation assays, we suggest that EVs-FMDVi could contribute substantially to the induction of an immune response against FMDVi. A remarkable proliferation was observed in B cells, more specifically in both, MZB and FoB cells subpopulations, indicating that viral proteins expressed on the surface of EVs are conformational antigens readily recognized by B cells through their BCR. An observation of note is that lymphocytes that have been primed *in vivo* via vaccination exhibit a reduced threshold for activation by EVs-FMDVi as compared to their naïve counterparts. This could be explained by the presence of memory cells. Due to their previous activation and selection in the germinal center, memory B cells are characterized by a lower response threshold, which allows them to respond faster and more powerfully than naïve B cells. Moreover, FoB lymphocytes showed a more significant increase in proliferation as compared to MZB. This could be attributed to the *in vivo* generation of memory FoB cells induced by immunization.

Lymphocyte activation is mainly based on three signals: the recognition of the antigen through the specific receptor, the signal from co-stimulatory molecules, and the last signal given by cytokines (43). EVs derived from DCs express in the membrane molecules that can provide some of these signals and make them small antigen presenting entities with the potential to activate T cells and B cells. T cell activation by EVs derived from APC is given by the interaction of the molecules present in the EVs with the complementary molecules present in the T cells (44). This work demonstrated that EVs-FMDVi induce the proliferation of previously primed T cells but not naïve T cells. As occurs with B cells, the proliferation of previously primed T cells by EVs-FMDVi would be given, mainly by memory T cells that were generated by the vaccination. However, FMDV-specific memory T cells should not be activated upon contact with EVs-LPS since these EVs, even though expressing high content of co-stimulatory molecules, do not present FMDV proteins associated with MHC that could provide the antigen-specific activation signal. Antigen-independent activation triggered by EVs-LPS could also be due to traces of LPS used to induce the maturation of APC *in vitro*. LPS coupled to EVs, by binding to their specific TLR, directly activates DCs, induces cytokine secretion, and consequently induces lymphocyte proliferation. However, the hypothesis of a possible mitogenic effect of EVs-LPS can be ruled out because they do not induce proliferation in naïve T cells. Furthermore, it is well known that B cells are stimulated in the presence of LPS. However, the absence of B cell activation by EVs-LPS suggests that any remnants of LPS that may have been left over from the purification of the vesicles were efficiently removed during this process. The ability of EVs-LPS to activate only previously sensitized T cells is more consistent with the hypothesis of bystander activation due to the high content of co-stimulatory molecules in EVs-LPS. In this case, the antigen-specific signal could be given by DC that endocyte small amounts of vaccine-antigen that persists within the spleen at day 14 post-vaccination (12, 45). This hypothesis is supported by data obtained from assays performed in naïve mice. These experiments revealed that the presence of EVs-LPS did not induce significant proliferation. These results suggest that in the absence of the initial activation signal (antigen recognition), co-stimulatory molecules expressed on EVs are insufficient to induce proliferation. The activation mediated by EVs derived from nonspecifically activated DCs was already demonstrated in another study (46). It was demonstrated that EVs obtained from POLI I:C activated DCs stimulate a tumor-specific response and induce neoplasia reduction when inoculated into tumor-bearing mice.

According to our results, EVs-FMDVi can trigger a B cell response but cannot activate T cells that have not previously been exposed to the antigen. In this study, we demonstrate that EVs derived from APC participate in B cell activation and could play an important role in the generation of the humoral response against FMDV.

Based on our findings, we hypothesize that EVs released *in vivo* by APC that have taken up the FMDVi vaccine antigen at the site of inoculation either could travel through the bloodstream or be secreted directly into the secondary lymph nodes. DCs could then take up the EVs-FMDVi and transport them to the T zone, where they would activate CD4+ and CD8+ T cells. Our research also reveals an interaction between EVs-FMDVi and MZB cells. These lymphocytes identify viral proteins/peptides carried by the EVs-FMDVi through the BCR and could transport them to the FDCs in the B zone. EVs would persist on the membrane of the FDCs, as what happens with infectious FMDV once the acute infection has subsided (47). This ongoing presence of antigen would aid in activating FoB, acting as a reservoir of natural or partially processed antigen.

Furthermore, our results show that FoB specifically recognizes viral proteins present on EVs-FMDVi. The viral antigens associated with the EVs membrane would facilitate BCR cross-linking, inducing endocytosis and subsequent activation of FoB. These cells would then process the viral proteins acquired with the EVs-FMDVi and present them to a T follicular helper (Tfh) in the T-B zone. The T-B collaboration would enhance the B cell response through cytokines secreted by the Thf. FoB would amplify the response through co-stimulatory molecules. Ultimately, the FoB would differentiate into antibody-secreting plasma cells and memory B cells. Thus, the EVs-FMDVi would help to amplify and generate the immune response induced by the FMDVi vaccine-antigen *in vivo*.

The results derived from this work describe for the first time that bone marrow derived APC co-cultured with FMDV vaccine-antigen secrete EVs expressing viral proteins/peptides capable of stimulating an FMDV-specific lymphocyte response *in vitro*. These findings provide relevant evidence to elucidate the poorly understood role of APC-derived EVs in B cell activation. APC-derived EVs would be a natural platform to present native or partially processed proteins that can remain longer in circulation. Being bound to membranes the pathogen proteins could be more stable (48); favoring BCR cross-linking and facilitating B cell activation. Not only would they serve as antigen carriers, but they would also actively participate in the generation and amplification of the immune response by activating MZB cells, FoB cells, and other DCs that have not yet been in contact with the antigen.

Research on EVs in veterinary science is still limited (49), but further study to understand their functions may be of great importance for animal health, diagnosis of diseases, and the generation of new therapies or improvement of existing ones. This work adds evidence to this field and proposes APC-derived EVs as a novel mediator of cellular communication between DC and lymphocytes in an anti-FMDV immune response. All our findings supply a more comprehensive understanding of the immune mechanisms triggered after FMDV vaccination, thus contributing to the rational design of novel vaccine platforms.

## Data availability statement

The raw data supporting the conclusions of this article will be made available by the authors, without undue reservation.

## Ethics statement

The animal study was approved by Comité Institucional para el Cuidado y uso de Animales de Experimentación (CICUAE)- Instituto Nacional de Tecnología Agropecuaria (INTA). The study was conducted in accordance with the local legislation and institutional requirements.

## Author contributions

FM: Investigation, Methodology, Writing – original draft, Writing – review & editing, Conceptualization, Formal analysis, Validation, Visualization. FC: Writing – review & editing, Formal analysis, Methodology, Visualization. MG: Data curation, Writing – review & editing, Formal analysis, Methodology. AE: Methodology, Writing – review & editing. JR: Writing – review & editing, Methodology. AF: Funding acquisition, Writing – review & editing, Methodology, Resources. CW: Writing – review & editing, Data curation, Formal analysis, Investigation, Supervision. CM: Conceptualization, Funding acquisition, Investigation, Resources, Supervision, Writing – original draft, Writing – review & editing, Formal analysis, Project administration, Validation.

## Supplementary material

Supplementary Figure 1 Characterization of APC differentiated from murine bone marrow precursors. **(A)** Graphs obtained by flow cytometry of cells harvested at day 8 of differentiation using commercial rGM-CSF factor are shown. **(B)** Two representative images (top and bottom) of confocal microscopy showing expression of CD11c protein labeled with specific monoclonal antibodies conjugated to APC are shown.

Supplementary Figure 2 Characterization of EVs by flow cytometry. Flow cytometry characterization of EVs isolated from APC using EVs and APC markers. EVs were coupled with aldehyde/sulfate latex beads of 4 µm diameter and incubated with different fluorochrome-conjugated monoclonal antibodies anti-CD9, anti-CD81, CD63, MHC-II, CD11c, CD86 as detailed in materials and methods. For viral protein detection on EVs, FITC-labeled IgG purified from an FMDV-immune bovine serum was used, and FITC-labeled IgG obtained from a healthy unimmunized bovine serum was used as a corresponding control (Control). Each histogram corresponds to one representative of fifteen independent experiments performed after each isolation round. Each histogram shows the control for bead’s autofluorescence (dotted line empty histogram), as well as the percentage of EVs positive for each marker (filled histograms). The analysis was performed in the *singlet region* determined from the SSC vs FSC light scatter plot.

## References

[1] Théry C Witwer KW Aikawa E Alcaraz MJ Anderson JD Andriantsitohaina R. Minimal information for studies of extracellular vesicles 2018 (MISEV2018): a position statement of the International Society for Extracellular Vesicles and update of the MISEV2014 guidelines. J Extracellular Vesicles. (2018) 7. doi: 10.1080/20013078.2018.1535750 PMC6322352 30637094

[2] Witwer KW Goberdhan DCI O’Driscoll L Théry C Welsh JA Blenkiron C. Updating MISEV: Evolving the minimal requirements for studies of extracellular vesicles. J Extracellular Vesicles. (2021) 10:e12182. doi: 10.1002/jev2.12182 34953156 PMC8710080

[3] Veerman RE Güçlüler Akpinar G Eldh M Gabrielsson S. Immune cell-derived extracellular vesicles – functions and therapeutic applications. Trends Mol Med. (2019) 25:382–94. doi: 10.1016/j.molmed.2019.02.003 30853173

[4] Buzas EI. The roles of extracellular vesicles in the immune system. Nat Rev Immunol. (2022) 23(4):236–50. doi: 10.1038/s41577-022-00763-8 PMC9361922 35927511

[5] Segura E Amigorena S Thery C. Mature dendritic cells secrete exosomes with strong ability to induce antigen-specific effector immune responses. Blood Cells Molecules Dis. (2005) 35:89–93. doi: 10.1016/j.bcmd.2005.05.003 15990342

[6] Admyre C Johansson SM Paulie S Gabrielsson S. Direct exosome stimulation of peripheral human T cells detected by ELISPOT. Eur J Immunol. (2006) 36:1772–81. doi: 10.1002/eji.200535615 16761310

[7] Qazi KR Gehrmann U Jordö ED Karlsson MCI Gabrielsson S. Antigen-loaded exosomes alone induce Thl-type memory through a B cell dependent mechanism. Blood. (2009) 113:2673–83. doi: 10.1182/blood-2008-04-153536 19176319

[8] Viaud S Terme M Flament C Taieb J André F Novault S. Dendritic cell-derived exosomes promote natural killer cell activation and proliferation: A role for NKG2D ligands and IL-15Rα. PloS One. (2009) 4:e4942. doi: 10.1371/journal.pone.0004942 19319200 PMC2657211

[9] Mattion N König G Seki C Smitsaart E Maradei E Robiolo B. Reintroduction of foot-and-mouth disease in Argentina: characterisation of the isolates and development of tools for the control and eradication of the disease. Vaccine. (2004) 22:4149–62. doi: 10.1016/j.vaccine.2004.06.040 15474705

[10] Borca MV Fernández FM Sadir AM Braun M Schudel AA. Immune response to foot-and-mouth disease virus in a murine experimental model: effective thymus-independent primary and secondary reaction. Immunology. (1986) 59:261. 3490436 PMC1453162

[11] Borca MV Garmendia AE Baxt B Moore DM Srikumaran S Morgan DO. Cross-reactive idiotopes among anti-foot and mouth disease virus neutralizing antibodies. Immunology. (1993) 79:368–74. PMC1421969 8406565

[12] López OJ Sadir AM Borca MV Fernández FM Braun M Schudel AA. Immune response to foot-and-mouth disease virus in an experimental murine model. II. Basis of persistent antibody reaction. Veterinary Immunol Immunopathology. (1990) 24:313–21. doi: 10.1016/0165-2427(90)90002-A 2160145

[13] Wigdorovitz A Zamorano P Fernández FM López O Prato-Murphy M Carrillo C. Duration of the foot-and-mouth disease virus antibody response in mice is closely related to the presence of antigen-specific presenting cells. J Gen Virol. (1997) 78:1025–32. doi: 10.1099/0022-1317-78-5-1025 9152419

[14] Galdo Novo S Malirat V Maradei ED Pedemonte AR Espinoza AM Smitsaart E. Efficacy of a high quality O1/Campos foot-and-mouth disease vaccine upon challenge with a heterologous Korean O Mya98 lineage virus in pigs. Vaccine. (2018) 36:1570–6. doi: 10.1016/j.vaccine.2018.02.015 29472132

[15] Abaracón D Giacometti H MJ. El uso de la etilenimina binaria (BEI) como inactivante de virus de la fiebre aftosa producido por diferentes técnicas semi-industriales. Bol Cent Panam Fiebre Aftosa. (1979) 33-34:1–5.

[16] Pega J Bucafusco D Giacomo S Schammas JM Malacari D Capozzo AV. Early adaptive immune responses in the respiratory tract of foot- and-mouth disease virus-infected cattle. J Virol. (2013) 87(5):2489–95. doi: 10.1128/JVI.02879-12 PMC3571376 23255811

[17] Dong Y Arif AA Poon GFT Hardman B Dosanjh M Johnson P. Generation and identification of GM-CSF derived alveolar-like macrophages and dendritic cells from mouse bone marrow. J Visualized Experiments. (2016) 112:1–7. doi: 10.3791/54194 PMC4993247 27404290

[18] Singh-jasuja H Thiolat A Ribon M Boissier M Bessis N Rammensee H. Immunobiology The mouse dendritic cell marker CD11c is down-regulated upon cell activation through Toll-like receptor triggering. Immunobiology. (2013) 218:28–39. doi: 10.1016/j.imbio.2012.01.021 22445076

[19] Harwood LJ Gerber H Sobrino F Summerfield A McCullough KC. Dendritic cell internalization of foot-and-mouth disease virus: influence of heparan sulfate binding on virus uptake and induction of the immune response. J Virol. (2008) 82:6379–94. doi: 10.1128/JVI.00021-08/ASSET/3AD1BAE8-0201-4A40-9F05-B2B39630921B/ASSETS/GRAPHIC/ZJV0130806910008.JPEG PMC2447102 18448534

[20] Tkach M Kowal J Zucchetti AE Enserink L Jouve M Lankar D. Qualitative differences in T-cell activation by dendritic cell-derived extracellular vesicle subtypes. EMBO J. (2017) 36:3012–28. doi: 10.15252/embj.201696003 PMC5641679 28923825

[21] Lamparski HG Metha-Damani A Yao JY Patel S Hsu DH Ruegg C. Production and characterization of clinical grade exosomes derived from dendritic cells. J Immunol Methods. (2002) 270:211–26. doi: 10.1016/S0022-1759(02)00330-7 12379326

[22] Cocozza F Martin-Jaular L Lippens L Di Cicco A Arribas YA Ansart N. Extracellular vesicles and co-isolated endogenous retroviruses from murine cancer cells differentially affect dendritic cells. EMBO J. (2023) 42:e113590. doi: 10.15252/embj.2023113590 38073509 PMC10711651

[23] Welsh JA Goberdhan DCI O’Driscoll L Buzas EI Blenkiron C Bussolati B. Minimal information for studies of extracellular vesicles (MISEV2023): From basic to advanced approaches. J Extracellular Vesicles. (2024) 13:e12404. doi: 10.1002/jev2.12404 38326288 PMC10850029

[24] De Toro J Herschlik L Waldner C Mongini C. Emerging roles of exosomes in normal and pathological conditions: new insights for diagnosis and therapeutic applications. Front Immunol. (2015) 6:255–89. doi: 10.3389/fimmu.2015.00203 PMC4418172 25999947

[25] Gorelik L Cutler AH Thill G Miklasz SD Shea DE Ambrose C. Cutting edge: BAFF regulates CD21/35 and CD23 expression independent of its B cell survival function. J Immunol. (2004) 172:762–6. doi: 10.4049/jimmunol.172.2.762 14707045

[26] Ostrowski M Vermeulen M Zabal O Geffner JR Sadir AM Lopez OJ. Impairment of thymus-dependent responses by murine dendritic cells infected with foot-and-mouth disease virus. J Immunol. (2005) 175:3971–9. doi: 10.4049/jimmunol.175.6.3971 16148145

[27] Ostrowski M Vermeulen M Zabal O Zamorano PI Sadir AM Geffner JR. The early protective thymus-independent antibody response to foot-and-mouth disease virus is mediated by splenic CD9 + B lymphocytes. J Virol. (2007) 81:9357–67. doi: 10.1128/JVI.00677-07 PMC1951431 17567692

[28] Bucio-López L Piñón-Zárate G Jarquín-Yáñezz K Hernández-Téllez B Herrera-Enríquez B. Phenotype of exosomes derived from dendritic cells treated with different stimuli. J Immunol Clin Res. (2018) 5(1):1046.

[29] Yáñez-Mó M Siljander PRM Andreu Z Zavec AB Borràs FE Buzas EI. Biological properties of extracellular vesicles and their physiological functions. J Extracellular Vesicles. (2015) 4:1–60. doi: 10.3402/jev.v4.27066 PMC4433489 25979354

[30] Colombo M Raposo G Théry C. Biogenesis, secretion, and intercellular interactions of exosomes and other extracellular vesicles. Annu Rev Cell Dev Biol. (2014) 30. doi: 10.1146/annurev-cellbio-101512-122326 25288114

[31] Sobrino F Domingo E. Foot and mouth disease foot and mouth disease current perspectives edited by. Boca Raton, Florida, USA: CRC Press (2004). doi: 10.1201/9781420037968

[32] Colino J Snapper CM. Exosomes from bone marrow dendritic cells pulsed with diphtheria toxoid preferentially induce type 1 antigen-specific igG responses in naive recipients in the absence of free antigen. J Immunol. (2006) 177:3757–62. doi: 10.4049/jimmunol.177.6.3757 16951336

[33] Näslund TI Gehrmann U Gabrielsson S. Cancer immunotherapy with exosomes requires B-cell activation. Oncoimmunology (2013) 2,6:e24533. doi: 10.4049/jimmunol.1203082 23894715 PMC3716750

[34] Zhang K Xu S Shi X Xu G Shen C Liu X Zheng H. Exosomes-mediated transmission of foot-and-mouth disease virus *in vivo* and *in vitro* . Vet Microbiol. (2019) 233:164–73. doi: 10.1016/J.VETMIC.2019.04.030 31176404

[35] Heath WR Kato Y Steiner TM Caminschi I. Antigen presentation by dendritic cells for B cell activation. Curr Opin Immunol. (2019) 58:44–52. doi: 10.1016/j.coi.2019.04.003 31071588

[36] Cinamon G Zachariah MA Lam OM Foss FW Cyster JG. Follicular shuttling of marginal zone B cells facilitates antigen transport. Nat Immunol. (2008) 9:54–62. doi: 10.1038/ni1542 18037889 PMC2488964

[37] Cerutti A Cols M Puga I. Marginal zone B cells: Virtues of innate-like antibody-producing lymphocytes. Nat Rev Immunol. (2013) 13:118–32. doi: 10.1038/nri3383 PMC3652659 23348416

[38] Kearney JF. Innate-like B cells. Springer Semin Immunopathology. (2005) 26:377–83. doi: 10.1007/s00281-004-0184-0 15645296

[39] Romero-Ramírez S Navarro-Hernandez IC Cervantes-Díaz R Sosa-Hernández VA Acevedo-Ochoa E Kleinberg-Bild A. Innate-like B cell subsets during immune responses: Beyond antibody production. J Leukocyte Biol. (2019) 105:843–56. doi: 10.1002/JLB.MR0618-227R 30457676

[40] Grasseau A Boudigou M Le Pottier L Chriti N Cornec D Pers JO. Innate B cells: the archetype of protective immune cells. Clin Rev Allergy Immunol. (2020) 58:92–106. doi: 10.1007/s12016-019-08748-7 31183788

[41] Weill JC Reynaud CA. IgM memory B cells: specific effectors of innate-like and adaptive responses. Curr Opin Immunol. (2020) 63:1–6. doi: 10.1016/j.coi.2019.09.003 31639539 PMC6942539

[42] Murphy K Weaver C. INMUNOBIOLOGY. In Janeway's Immunbiology. (2017). doi: 10.1007/s13398-014-0173-7.2

[43] Cantrell D. Signaling in lymphocyte activation. Cold Spring Harbor Perspect Biol. (2015) 7:1–14. doi: 10.1101/cshperspect.a018788 PMC4448613 26032717

[44] Kowal J. Dendritic cell extracellular vesicles. In: Immunobiology of dendritic cells part B, 1st ed, vol. 349. Amsterdam, Netherlands: Elsevier Inc (2019). doi: 10.1016/bs.ircmb.2019.08.005

[45] Wigdorovitz A Carrillo C Dus Santos MJ Trono K Peralta A Gómez MC. Induction of a protective antibody response to foot and mouth disease virus in mice following oral or parenteral immunization with alfalfa transgenic plants expressing the viral structural protein VP1. Virology. (1999) 255:347–53. doi: 10.1006/viro.1998.9590 10069960

[46] Sobo-Vujanovic A Munich S Vujanovic NL. Dendritic-cell exosomes cross-present Toll-like receptor-ligands and activate bystander dendritic cells. Cell Immunol. (2014) 289:119–27. doi: 10.1016/j.cellimm.2014.03.016 PMC4045011 24759079

[47] Gordon L Mabbott N Charleston B Perez E. Identifying the role of complement receptor 2 (CR2) on follicular dendritic cells (FDCs) in the persistence of foot and mouth disease virus (FMDV). Access Microbiol. (2019) 1:201. doi: 10.1099/acmi.ac2019.po0074

[48] Ruan S Greenberg Z Pan X Zhuang P Erwin N He M. Extracellular vesicles as an advanced delivery biomaterial for precision cancer immunotherapy. Advanced Healthcare Materials. (2021) 11(5):2100650. doi: 10.1002/adhm.202100650 PMC8720116 34197051

[49] Xiong Y Lou P Xu C Han B Liu J Gao J. Emerging role of extracellular vesicles in veterinary practice: novel opportunities and potential challenges. Front Veterinary Sci. (2024) 11:1335107. doi: 10.3389/fvets.2024.1335107 PMC10850357 38332755

