# Supplementary figures and images for: Extracellular vesicles derived from antigen-presenting cells pulsed with foot and mouth virus vaccine-antigens act as carriers of viral proteins and stimulate B cell response
Source: Front Immunol. 2024 Aug 8;15:1440667. doi: 10.3389/fimmu.2024.1440667 (PMC11338771; doi:10.3389/fimmu.2024.1440667)

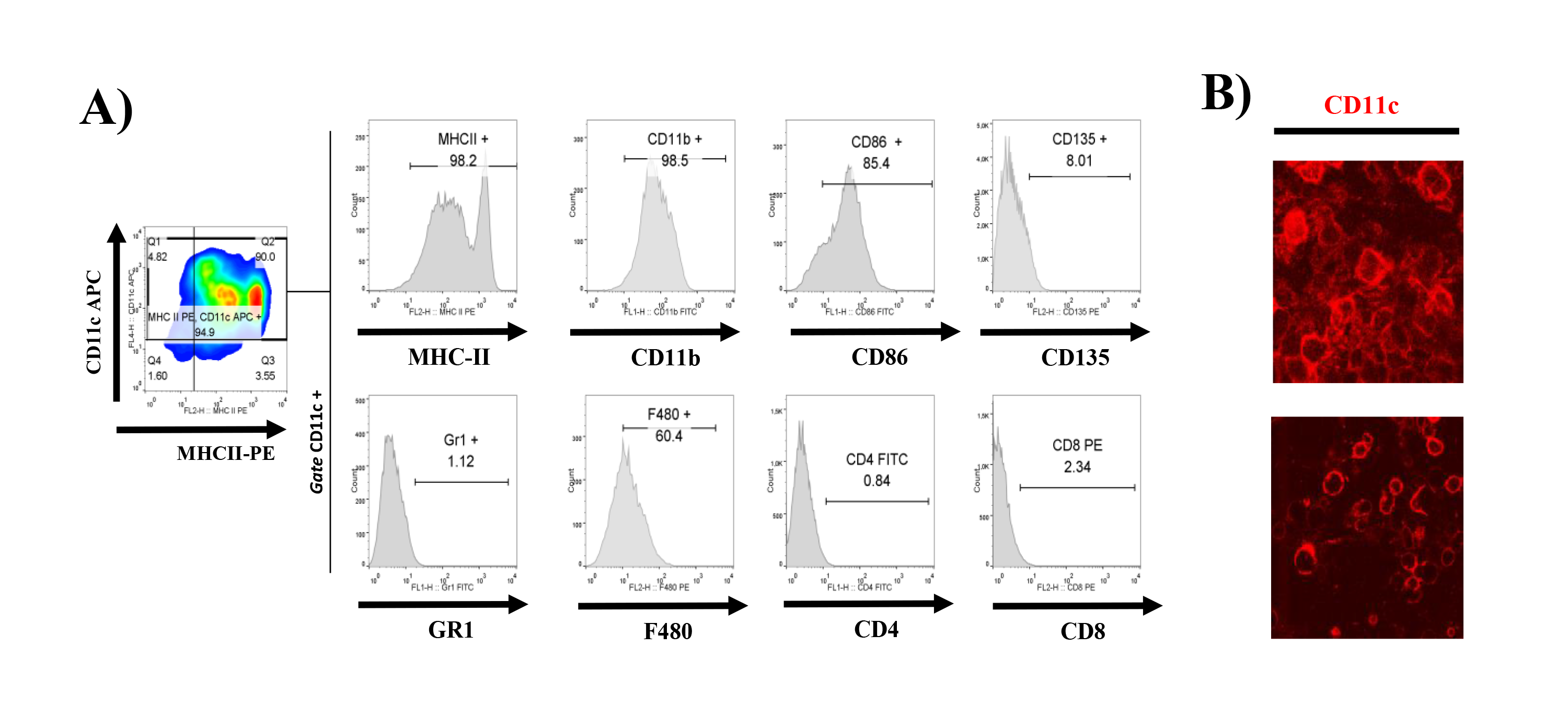

Supplement: Supplementary Figure 1 — Characterization of APC differentiated from murine bone marrow precursors. (A) Graphs obtained by flow cytometry of cells harvested at day 8 of differentiation using commercial rGM-CSF factor are shown. (B) Two representative images (top and bottom) of confocal microscopy showing expression of CD11c protein labeled with specific monoclonal antibodies conjugated to APC are shown. [file Image_1.tif]

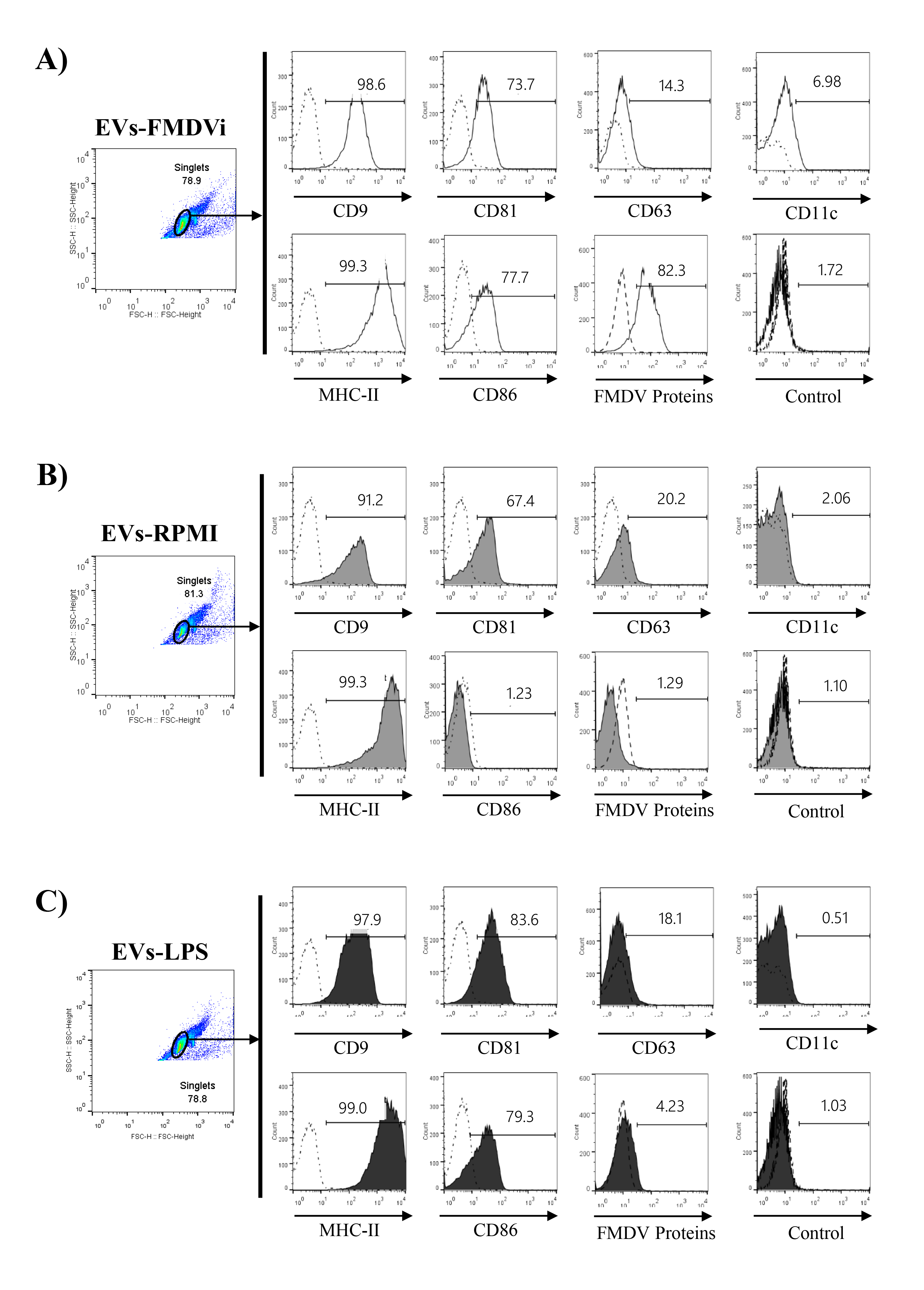

Supplement: Supplementary Figure 2 — Characterization of EVs by flow cytometry. Flow cytometry characterization of EVs isolated from APC using EVs and APC markers. EVs were coupled with aldehyde/sulfate latex beads of 4 µm diameter and incubated with different fluorochrome-conjugated monoclonal antibodies anti-CD9, anti-CD81, CD63, MHC-II, CD11c, CD86 as detailed in materials and methods. For viral protein detection on EVs, FITC-labeled IgG purified from an FMDV-immune bovine serum was used, and FITC-labeled IgG obtained from a healthy unimmunized bovine serum was used as a corresponding control (Control). Each histogram corresponds to one representative of fifteen independent experiments performed after each isolation round. Each histogram shows the control for bead’s autofluorescence (dotted line empty histogram), as well as the percentage of EVs positive for each marker (filled histograms). The analysis was performed in the singlet region determined from the SSC vs FSC light scatter plot. [file Image_2.tif]
